# Applying whole-genome and whole-exome sequencing in breast cancer: a review of the landscape

**DOI:** 10.1007/s12282-024-01628-9

**Published:** 2024-08-27

**Authors:** Hetvi Ganatra, Joecelyn Kirani Tan, Ana Simmons, Carola Maria Bigogno, Vatsala Khurana, Aruni Ghose, Adheesh Ghosh, Ishika Mahajan, Stergios Boussios, Akash Maniam, Olubukola Ayodele

**Affiliations:** 1 Barts Cancer Institute, Cancer Research UK City of London, Queen Mary University of London, London, UK; 2 School of Medicine, University of St. Andrews, Fife, Scotland, UK; 3 Andrews Oncology Society, Scotland, UK; 4 Faculty of Biology, Medicine and Health, University of Manchester, Manchester, UK; 5 Department of Medical Oncology, Barts Cancer Centre, St Bartholomew’s Hospital, Barts Health NHS Trust, London, UK; 6 British Oncology Network for Undergraduate Societies (BONUS), London, UK; 7 William Harvey Research Institute, Queen Mary University of London, London, UK; 8 Department of Medical Oncology, Medway NHS Foundation Trust, Gillingham, Kent UK; 9 Department of Medical Oncology, Mount Vernon Cancer Centre, Mount Vernon and Watford NHS Trust, Watford, UK; 10 UCL Cancer Institute, University College London, London, UK; 11 Department of Oncology, Lincoln Oncology Centre, Lincoln County Hospital, United Lincolnshire Hospitals NHS Trust, Lincoln, UK; 12 Faculty of Life Sciences & Medicine, School of Cancer & Pharmaceutical Sciences, King’s College London, London, UK; 13 Kent and Medway Medical School, University of Kent, Canterbury, Kent UK; 14 Faculty of Medicine, Health, and Social Care, Canterbury Christ Church University, Canterbury, UK; 15 AELIA Organization, 9th Km Thessaloniki–hermi, 57001 Thessaloniki, Greece; 16 Department of Medical Oncology, Portsmouth Hospitals University NHS Trust, Portsmouth, UK; 17 Faculty of Science and Health, School of Pharmacy and Biomedical Sciences, University of Portsmouth, Portsmouth, UK; 18 Caribbean Cancer Research Institute, Port of Spain, Trinidad and Tobago; 19 Department of Medical Oncology, University Hospitals of Leicester NHS Trust, Leicester, UK; 20 Leicester Cancer Research Centre, University of Leicester, Leicester, UK

**Keywords:** Sequencing, Breast cancer, Exome, Genome, Whole-genome sequencing, Whole-exome sequencing

## Abstract

Whole-genome sequencing (WGS) and whole-exome sequencing (WES) are crucial within the context of breast cancer (BC) research. They play a role in the detection of predisposed genes, risk stratification, and identification of rare single nucleotide polymorphisms (SNPs). These technologies aid in the discovery of associations between various syndromes and BC, understanding the tumour microenvironment (TME), and even identifying unknown mutations that could be useful in future for personalised treatments. Genetic analysis can find the associated risk of BC and can be used in early screening, diagnosis, specific treatment plans, and prevention in patients who are at high risk of tumour formation. This article focuses on the application of WES and WGS, and how uncovering novel candidate genes associated with BC can aid in treating and preventing BC.

## Introduction

Breast cancer (BC) stands as a significant global health challenge. Prior to 2020, BC ranked as the second most prevalent cancer worldwide amongst females; however, since 2020, it has surpassed lung cancer in prevalence, marking a notable shift in disease burden [1]. In 2020 alone, 2.3 million new cases of BC were diagnosed in women, resulting in 685,000 deaths globally, rendering it the fifth leading cause of death that year [2]. Such statistics underscore the urgent need to address the burden of BC. Twenty-three percent of BC cases in the UK can be preventable [3]. Thus, emphasis on prevention as well as optimal treatment becomes paramount.

BC diagnosis typically involves a multifaceted approach. First, clinical breast examination (CBE) conducted by healthcare professionals entails a physical assessment of the breasts for anomalies such as lumps, changes in size or shape, or skin dimpling [4, 5]. Mammography, an X-ray imaging technique, aids in detecting suspicious masses or calcifications in breast tissue which are otherwise not found or fully interpreted by CBE. Ultrasound, another imaging technique which utilises sound waves, offers further imaging to distinguish between solid masses and fluid-filled cysts [5, 6]. Lastly, if anything is suspected, a biopsy can be taken and thoroughly analysed in a laboratory.

Treatment modalities for BC encompass pharmacological, radiological, and surgical interventions. Pharmacological approaches involve chemotherapy, hormone therapy, and/or targeted therapy [7].

Radiological treatments target BC cells and surrounding lymph nodes, while surgical options include lumpectomy or mastectomy to remove tumours or the entire breast tissue [8–10]. These management options can also be utilised in combination for optimal cancer management. Whole-genome sequencing (WGS) and whole-exome sequencing (WES) have emerged as technologies capable of elucidating genetic underpinnings in diseases [11]. Understanding the genetic landscape of BC is imperative, with genetic testing identifying individuals harbouring inherited mutations such as *BRCA1* and *BRCA2*, which substantially elevate BC risk [12]. This comprehension facilitates targeted screening and preventive measures, such as risk-reduction surgery or intensified surveillance, in high-risk individuals and their relatives. Moreover, genetic biomarkers enable early BC detection and administration of preventive interventions. Identification of causal genes or genes predisposing to BC aids in tailored treatment provision, fostering the development of personalised medicine. Thus, this paper aims to delineate the applications of WES and WGS in the discovery of important biomarkers in BC.

## Breast cancer

The two most prevalent types of BC are invasive ductal carcinoma (IDC) and invasive lobular carcinoma (ILC), although other types based on origin of cancer cells have been highlighted in Table 1 [13]. Phyllodes tumours of the breast are rare fibroepithelial tumours, constituting approximately 0.3–0.5% of all primary breast tumours [14]. Furthermore, according to molecular subtypes, BC can be divided into four major forms as shown in Table 2 [15]. The various types of division become necessary for identifying optimal and personalised treatment plans for each patient.

**Table 1:** Different types of BC based on origin of the cancerous cells

	Description
Type of BC	
Invasive ductal carcinoma (IDC)	Originates in the ducts of the breast and spreads rapidly through invasion and metastasis to other parts of the breast and body
Invasive lobular carcinoma (ILC)	Begins in the lobules of the breast and advances to nearby organs through invasion and metastasis
Inflammatory breast carcinoma	Characterised by inflammation leading to redness, swelling, and tenderness of the breast
Metastatic BC	Cancer spreads to distant organs such as lungs, liver, or bones
Male BC	Rare, affecting about 1% of BC cases, primarily in older men
Other subtypes	
Paget’s disease	Affects the skin of the nipple and areola, often accompanied by an underlying BC
Medullary ductal carcinoma	Tumour cells resemble the medulla (inner part) of the brain
Mucinous ductal carcinoma	Tumour cells produce mucus, often forming distinct mucus-filled cysts
Papillary ductal carcinoma	Tumour cells grow in finger-like projections, often detected through mammograms or ultrasound
Tubular ductal carcinoma	Tumour cells resemble small tubes or ducts under the microscope

*BC* breast cancer

**Table 2:** Molecular subtype of BC

Type	Subtype	Cases
Luminal A	ER/PR + , HER2 – [low ki67 (≤ 13.25%)]	70–80%
Luminal B	ER/PR + , HER2 + [high ki67 (> 13.25%)]	10–15%
HER2 +	ER/PR –, HER2 +	4–10%
TNBC (basal-like)	ER/PR–, HER2–	10–15%

*ER* oestrogen, *PR* progesterone, *HER2* human epidermal growth factor receptor 2, ki67, cell proliferating factor, *BC* breast cancer, *TNBC* triple-negative breast cancer

### Aetiology

Most BC cases are sporadic, caused by smoking, alcohol consumption, obesity, pregnancy, breast density, age, serum estradiol level, oral contraceptive, exposure to radiation, environmental pollutants and more. Women are at an even higher risk of carcinogenic effects on their gonadal hormone during menarche and menopause [16]. Apart from this, previous history of fibrocystic diseases can also be a risk factor for developing BC [17]. However, hereditary and germline mutations account for 8–10% of all BC, and 50% of these cases are detected with germline *BRCA1/2* mutations, while the rest have moderate penetrance rare genes or common but low penetrance genes mutations [11].

### Pathophysiology

**Fig. 1:**
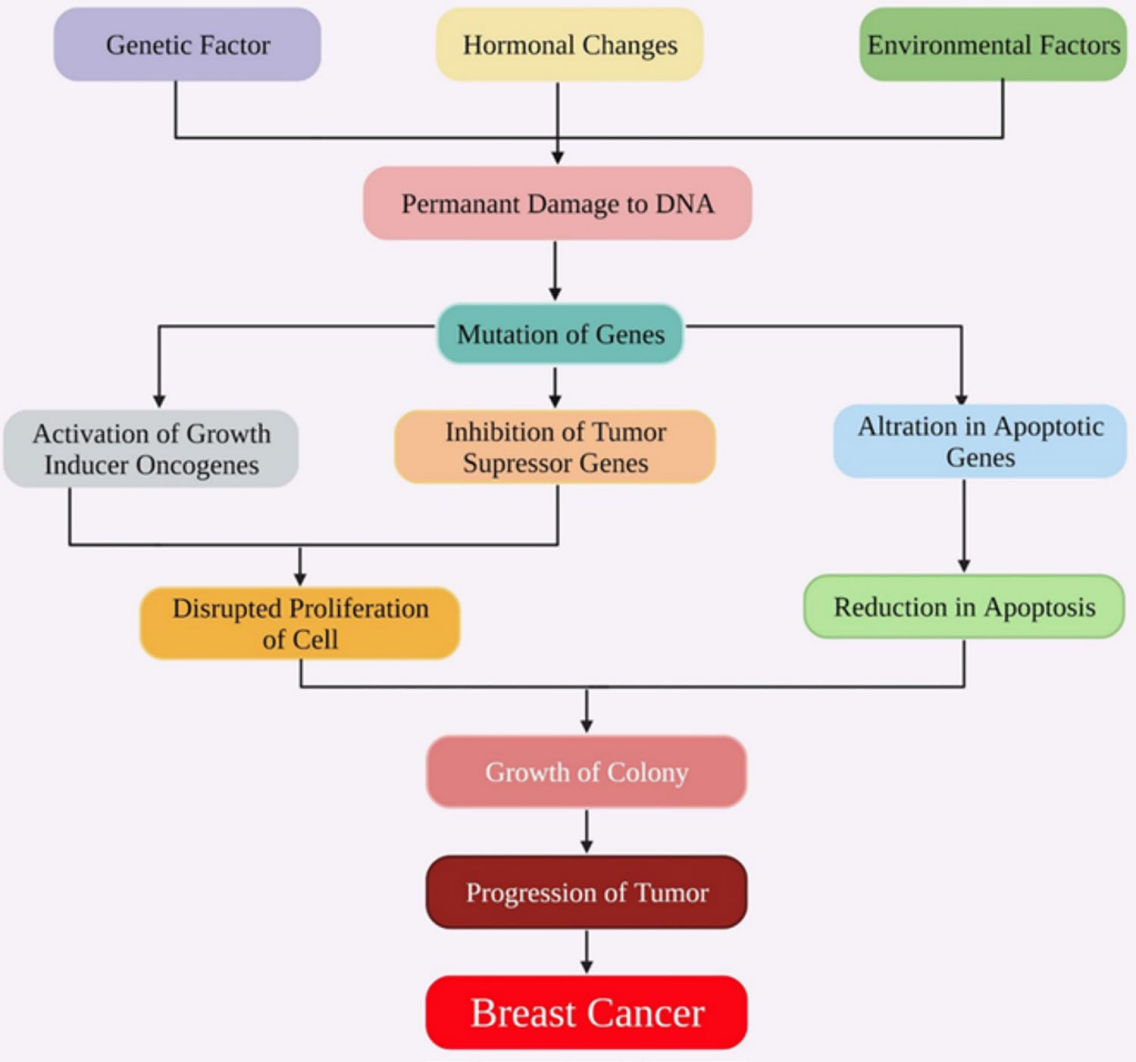
Pathophysiology of BC in association with risk factors (Alharbi et al. 2022)

The environmental, genetic, and hormonal factors highlighted above ultimately damage the DNA, causing both genetic and epigenetic changes. These changes promote dysregulation of cells and induce aberrant cell progression, leading to breast cancer (BC) (Fig. 1).

## Genes responsible for breast cancer

BC is a complex and heterogeneous disease; there are known to be around 30 genes which are associated risk factors of BC [18, 19]. These genes include predisposed genes, high, moderate, and low penetrance genes, syndrome-associated genes, single nucleotide polymorphism and some common variants, which are likely to be mutated and be a precursor for BC [16].

**Fig. 2:**
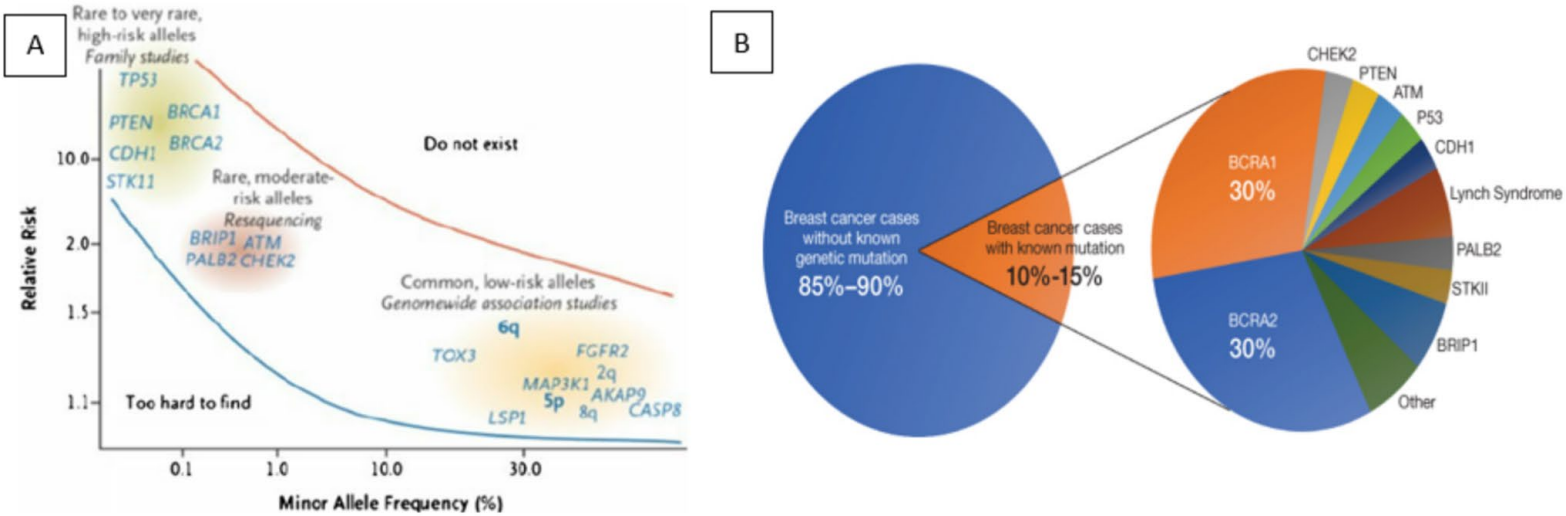
**A** BC susceptibility loci and genes. **B** Gene associated with BC

The high penetrance genes, in the germline mutation such as *BRCA1*, *BRCA2*, *CDH1*, *PALB2*, *PTEN* and *TP53*, confer significant risk of developing carcinoma [20], whereas moderate penetrance genes such as *PABL2* and *ATM* contribute moderate risk, and low penetrance genes such *SNPS* and *MAP3K1* are associated with low elevated risk [16] (Fig. 2A). These genes and the associated risk percentages are highlighted in Table 3.

**Table 3:** Genes associated with breast cancer and their risks

Type	Gene	Effects/functions	Risk
High penetrant	*BRCA 1* (autosomal dominant)	Tumour suppressor gene—involved in breaking dsDNA	Female—50–85% Male—5–10%
	*BRCA2* (autosomal dominant)	Tumour suppressor gene—involved in breaking dsDNA, homologous recombination	Female—50–85%
	*PTEN*	Cowden syndrome	85%
	*TP53*	Li–Fraumeni syndrome	25%
	*CDH1*	Hereditary diffuse gastric cancer	39%
	*STK11*	Peutz–Jeghers syndrome	32%
Moderate penetrant (rare)	*CHEK2*	DNA repair	RR—1.70
	*BRIP1*	Interacts with the BRCA1 C-Terminus (BRCT) domain of BRCA1	RR—2.0
	*ATM*	Monitoring and DNA repair	RR—2.37
	*PALB2*	Nuclear localisation	
	*RAD51C*	Recombinational repair of ds DNA breaks	
Low penetrant (common)	*MAP3K1*	Cell growth, proliferation	5–10% (mostly in ER + /PR +)
	*LSP1*	Cell motility and adhesion	
	*TERT*	Chromosome telomere	
	*CASP8*	Apoptosis	
	*TOX3*	Chromatin-associated protein	1.64-fold homozygous risk (especially in ER +)
	*RBL2*	Cell cycle regulation	
	*CDKN2A/B*	Cyclin-dependent kinase	
	*MYEOV/CCNDL*	Fibroblast growth factors	
	*ZNF365*	Zinc finger protein	
	*ANKRD16/FBXO18*	Helicase	
	*ZMIZ1*	Transcription factors	
Single nucleotide polymorphism (SNPs)	*FGFR2*	Cell growth and proliferation	
	*FGF-8*	Cell growth and proliferation	
	*8q24* (no known gene)	Enhancer of MYC, protooncogene	
	*2q35* (no known gene)		
	*LSP1*	F-actin binding cytoskeletal protein	
	*SLC4A7 or NEK10 (3p24)*		OR—1.11 and 0.97 for heterozygote and homozygote genotypes, respectively
	*MRPS30 (5p12)*		Mostly ER +

*BC* breast cancer, *BRCA1* breast cancer gene 1, *BRCA2* breast cancer gene 2, *PTEN* phosphatase and tensin homolog, *TP53* tumour protein p53, *CDH1* cadherin-1, *STK11* serine/threonine kinase 11, *CHEK2* checkpoint kinase 2, *BRIP1* BRCA1-interacting protein C-terminal helicase 1, *ATM* ataxia telangiectasia mutated, *PALB2* partner and localiser of BRCA2, *RAD51C* RAD51 homolog C, MAP3K1 mitogen-activated protein kinase kinase kinase 1, *LSP1* lymphocyte-specific protein 1, *TERT* telomerase reverse transcriptase, *CASP8* caspase 8 *TOX3* TOX high mobility group box family member 3, *RBL2* retinoblastoma-like protein 2, *FGFR2* fibroblast growth factor receptor 2, *FGF-8* fibroblast growth factor 8, *LSP1* lymphocyte-specific protein 1, *SLC4A7* solute carrier family 4 member 7, *NEK10* NIMA (never in mitosis gene A)-related kinase 10, *MRPS30* mitochondrial ribosomal protein S30, *RR* relative risk, *OR* odds ratio

The collective contribution of identified BC genes currently only constitutes approximately 30% of the risk associated with BC, leaving a substantial portion of the genetic factors underlying the disease still unknown, as depicted in Fig. 2B, where 85–95% of genetic mutations responsible for cancer remain unidentified. To reduce the risk of occurrence and increase the survival rate of BC patients, it is of paramount importance to uncover these missing genetic elements. We have highlighted the transition in genetic testing methods for BC as below.

### Early genetic testing methods

In the early 1990s, cytogenetic techniques were used to detect chromosomal alterations in BC tumour-derived cultures compared to normal tissues [21]. Through chromosome counts and karyotyping, significant numerical changes and structural abnormalities were detected, including alterations in chromosomes 17, 18, 20 and 21, as well as structural rearrangements such as translocations and inversions. However, cytogenetic analysis has limitations, particularly in cases of complex karyotypes where interpretation becomes challenging [22].

Later developments in genetic testing for BC included the discovery of the *BRCA1* gene by a team at the Institute of Cancer Research in London in 1994, establishing its clinical implications [23, 24]. This discovery highlighted that the *BRCA1* gene is inherited, leading to testing focussing on identifying mutations in specific genes associated with hereditary BC, such as *BRCA1* and *BRCA2* [24].

As research progressed, scientists recognised the complexity of BC genetics and the contribution of multiple genetic factors to disease susceptibility. This realisation led to the development of more comprehensive testing approaches, such as RNA-sequencing, sequencing of methylated DNA, WES and WGS [25]. In addition, recent research has focussed on integrating genomic data with other *-omics* technologies, such as transcriptomics and epigenomics, to gain a more comprehensive understanding of BC biology. Integrative analyses have provided insights into the molecular mechanisms driving BC heterogeneity and identified potential therapeutic targets [26].

### Advanced genetic testing methods

The human genome consists of coding, noncoding regions, and pseudogenes. Eighty-five percent of disease-causing mutations are present in the functional coding region of the genome. In the late 2000s and early 2010s, WES was used for sequencing protein-coding regions of the genome, known as exome as well as splice-site variants to potentially uncover rare genes, predisposing variants, and genetic disorders. After the completion of the Human Genome Project in 2003, it was recognised that many variants are present in the intergenic regions—introns and noncoding regions—which can also cause epigenetic changes in cancer [27]. This can be sequenced by WGS [28].

Unlike earlier genetic testing, which focussed on a predefined set of genes, WES and WGS provide a comprehensive view of BC genetics. These advanced technologies are essential for the comprehensive analysis of complex cancer. They are less expensive in terms of sequencing cost per genome/exome, more accurate, and provide individualised treatment plans [28].

## Application of WES and WGS

WES and WGS are revolutionising our ability to identify novel genetic variants associated with cancer predisposition [20]. Thus, researchers can offer a solid basis for diagnosis, therapy, as well as prevention for BC patients with the aid of next-generation sequencing (NGS), i.e. WES and WGS [19]. There are several applications of these techniques, including early diagnosis, prevention, analysis of recurrence, treatment, and research.

### Early diagnosis

These sequencing techniques help to identify the unique genetic makeup of a patient with a significant genetic history, uncovering heritability genes that contribute alleles segregating in an autosomal dominant pattern. For instance, testing for inherited *BRCA1/2* genes from the blood samples of patients allows for adequate risk management and care. This testing can guide decisions on risk-reduction strategies, such as total mastectomy and/or bilateral oophorectomy, and serves as a companion diagnostic for PARP inhibitors among patients. For family relatives carrying the same pathogenic variant, single-site PCR methods can be employed for monitoring and surveillance against cancer occurrence until risk-reduction surgery is performed.

WGS can also be applied to noncoding regions of the genome to find low penetrance genes, such as SNPs, in the intergenic regions. These low penetrance genes can cause variation in gene regulatory elements, promoting growth and leading to formation of cancer, which can be detected early [19]. Similarly, cancer-associated genes are enriched in differentially methylated regions (DMRs) present in the introns undergoing epigenetic changes. For instance, the *PAX5* can become hypermethylated, causing anti-suppressor activity, while the *PAX6* can become hypomethylated, causing upregulated expression to promote cancer [29].

### Prevention

WGS and WES provide insights into detecting highly susceptible genes or syndromes associated with increased risk of BC, which can in turn drive further measures to prevent the occurrence of diseases by modifying lifestyles or increasing frequencies of screening.

In a study from Hamdi et al., WES was performed using family-based approach, thus the shared variation in exome in family could be pre-detected giving a higher chance to reduce the risk of occurrence within the family. In the study, four genes were identified (*XRCC2*, *MAPKAP1*, *FANCM* and *RINT1*) with BC risk which seem to be inherited within the family in a specific manner [29]. Initially, the same study was performed by traditional NGS which led to identification of BRCA genes but not of the other susceptible gene [30].

Furthermore, studies show that there is increased lifetime risk of almost 50% of lobular breast carcinoma associated with the germline mutation of *CDH1* gene, which are primary linked to hereditary diffuse gastric cancer (HDGC) without any mutation in *BRCA1/2* gene [31]. Such various hereditary syndromes associated with increased risk of BC can be identified and treated.

### Diagnosis after occurrence

WES and WGS play an integral role in the post cancer diagnosis phase, as the detailed tumour profiling can assist the clinicians to identify targetable mutations, to discuss various therapeutic approaches and to assess long-term risk of second primary tumour. A study carried out using NGS on an Asian population with *BRCA*-negative BC patients showed variants that were nonsynonymous single nucleotide variants (SNVs) (85.7%). The study was compared with a US-based case cohort and found 14 variants that were consistently enriched in the patient cohort. Seven variants were further explored and confirmed with Sanger sequencing (*GPRIN2*, *NRG1*, *MYO5A*, *CLIP1*, *CUX1*, *GNAS*, and *MGA*) [20]. This study recognised that SNVs can be used as biomarkers and that these mutations help unravel the genetic landscape of BC and provide insights into the genes and pathways driving the disease [20]. In addition, tumour-infiltrating lymphocytes (TILs) and programmed cell death protein 1 (*PD-1*) can also serve as predictive biomarkers in advanced triple-negative BC (TNBC) [11].

### Treatment

WES and WGS enable comprehensive genetic profiling of both the tumour and the patient, facilitating risk stratification between patients with variable genetic makeup, and personalised treatment approaches in BC. By integrating genetic information with clinical and pathological data, healthcare providers can optimise treatment strategies to improve patient outcomes.

#### Chemotherapy

WES and WGS identify specific mutations that can be used to detect the sensitivity of a chemotherapy drug towards specific regions, resulting in higher efficacy. In the context of BC, homologous recombination deficiency (HRD) has been identified as a key factor in determining sensitivity to certain chemotherapeutic agents, such as platinum-based chemotherapy and poly (ADP-ribose) polymerase (PARP) inhibitors [32].

To optimise dosages for patients, methods have been developed to reliably detect HRD status. Initially, HRD status focussed solely on the detection of HRD-associated variants, but using WGS identification of characteristic genomic damage patterns induced by HRD, including genome-wide loss of heterozygosity (LOH), telomeric allelic imbalance (TAI), and large-scale state transition (LST) were recognised. Furthermore, genomic mutational signatures have proven useful in HRD detection [33]. Clinical trials have also demonstrated that the predictive value of HR deficiency can determine the response of patients towards neoadjuvant platinum-based chemotherapy. Specifically, HR deficiency has been associated with higher rates of complete response and lower residual cancer burden scores. Moreover, HR deficiency has been shown to identify TNBC tumours, including those without *BRCA1/2* mutations that are more likely to respond to platinum-containing therapy [34].

In addition to the relationship between HRD and platinum-based therapies, various cytotoxic drugs used in BC treatment have pharmacogenomic associations. Anthracyclines, for example, exert their effects by modulating the expression of genes associated with the regulation of natural killer cell-mediated cytotoxicity and the JAK-STAT signalling pathway. These genes, which can be identified by WES or WGS, help assess the efficacy of anthracyclines like doxorubicin [35]. The pharmacogenomic information for other cytotoxic drugs, such as 5-fluorouracil (5-FU) and irinotecan, also play a critical role in personalised BC treatment. For 5-FU, the dihydropyrimidine dehydrogenase (DPYD) enzyme and thymidylate synthase (TYMS) are key pharmacogenomic markers. Similarly, irinotecan's efficacy and toxicity are influenced by the UGT1A1 gene, which encodes the enzyme UDP-glucuronosyltransferase involved in its metabolism.to the relationship between HRD and platinum-based therapies, various cytotoxic drugs used in BC treatment have pharmacogenomic associations. Anthracyclines, for example, exert their effects by modulating the expression of genes associated with the regulation of natural killer cell-mediated cytotoxicity and the JAK-STAT signalling pathway [35]. These genes, which can be identified by WES or WGS, help assess the efficacy of anthracyclines like doxorubicin [35]. The pharmacogenomic information for other cytotoxic drugs, such as 5-fluorouracil (5-FU) and irinotecan, also play a critical role in personalised BC treatment. For 5-FU, the dihydropyrimidine dehydrogenase (DPYD) enzyme and thymidylate synthase (TYMS) are key pharmacogenomic markers. Similarly, irinotecan’s efficacy and toxicity are influenced by the UGT1A1 gene, which encodes the enzyme UDP-glucuronosyltransferase involved in its metabolism.

In summary, WGS offers a comprehensive approach to identifying HRD status and predicting responses to specific chemotherapeutic agents in BC. The integration of pharmacogenomic information for various cytotoxic drugs, such as anthracyclines, 5-FU, and irinotecan, enhances the precision of personalised treatment plans, ultimately improving patient outcomes.

#### Immunotherapy

WES and WGS identify mutations and direct the immune cells towards it. A WES study analysing metastatic tumour from 37 BC patients observed nonsynonymous somatic mutations across the whole patient cohort. Moreover, to study the immune response within the BC tumour microenvironment (TME), researchers successfully grew tumour-infiltrating lymphocytes (TILs) ex vivo from these tumours. Functional assays were then conducted to evaluate the reactivity of autologous TILs against mutated proteins within the tumours. These assays were indication of T cell activation and effector function response in response to antigen recognition and the results from the study revealed that autologous TILs recognised at least one mutated protein in 25 out of 37 patients (68%). Furthermore, 75% of the recognised mutated proteins were identified by CD4 + T cells, while 25% were identified by CD8 + T cells, which indicates the involvement of both CD4 + helper T cells and CD8 + cytotoxic T cells in recognising and targeting tumour-specific mutations. All identified immunogenic tumour mutations were unique to each patient, which highlights the importance of individualised immunotherapy for BC patients [36].

#### Endocrine therapy

Endocrine therapy involves the identification of specific mutations in molecular subtypes which will subsequently help in administration of hormonal therapy tailored to these mutations. In another study which uses WES, significantly different mutation patterns were observed among luminal A, luminal B, basal-like, and HER2-enriched (HER2E) subtypes. Luminal A had the *PIK3CA* mutation identified most frequently, followed by *MAP3K1*, *GATA3*, *TP53*, *CDH1*, *and MAP2K4*. Inactivating mutations in *MAP3K1* and *MAP2K4* were noticed, which are key components of the *p38–JNK1* stress kinase pathway, indicating potential sensitivity to targeted therapies against this pathway. On the other hand, TP*53* and *PIK3CA* mutations were observed in luminal B cancers. *HER2* was characterised by frequent *HER2* amplification and showed a hybrid mutation pattern with high frequencies of *TP53* and *PIK3CA* mutations [26].

Overall, the integration of WES and WGS data enables the identification of subtype-specific mutation patterns, guiding the selection of optimal endocrine therapy regimens tailored to the molecular characteristics of individual BC patients, thereby improving treatment efficacy and patient outcomes. For instance, tamoxifen represents a primary endocrine therapy for BC cases characterised by ER/PR-positive phenotypes [37].

#### Targeted treatment

WGS has exhibited effectiveness in pinpointing tumorigenic drivers linked to chromothripsis in BC [38]. Chromothripsis is characterised by catastrophic genomic rearrangements and is affecting over 60% of metastatic BC cases and 25% of luminal BC. After analysis of chromothripsis events, sequencing revealed alterations in multiple chromosomes, particularly chromosomes 11 and 17, harbouring significant driver genes such as *CCND1*, *ERBB2*, *CDK12*, and *BRCA1*. Moreover, chromothripsis leads to the formation of recurrent fusion genes that drive tumour progression [39]. Within genomic regions highly susceptible to oestrogen receptor alpha (ERɑ)-related chromothripsis, a subgroup of genes, including Tousled-Like Kinase 2 (*TLK2*), has been identified as upregulated in tumours exhibiting chromothripsis [38]. Furthermore, research indicates distinct patterns of genomic instability among clinical subtypes, with chromothripsis significantly contributing to instability in high-risk breast tumours. Notably, chromosome 17 emerges as the most frequently affected chromosome across all subtypes, suggesting a non-random, stepwise pattern of genomic instability targeting specific chromosomes [40]. Thus, phenothiazine antipsychotics (PTZs) have emerged as promising adjuncts for treatment due to their antiproliferative effects in BC cell lines and patient-derived circulating BC cells [38].

### Towards clinical trials

WES and WGS serve as invaluable tools for advancing our understanding of BC biology, offering a comprehensive perspective encompassing genetic, epigenomic, and functional attributes. These technologies hold significant promise for research endeavours focussing on BC.

Ethnicity influences the incidence of TNBC, with African American and Hispanic women displaying a heightened risk compared to other ethnicities. In addition, African American women tend to exhibit poorer prognoses in comparison to other ethnic groups [41]. However, the precise underlying mechanisms driving these disparities remain largely obscure [42]. Nevertheless, WGS and WES present promising avenues for elucidating potential genetic predispositions that may contribute to these observed trends. In pharmacogenetics research, the identification of genetic variants influencing protein activity within pathways can inform assessments of drug efficacy and toxicity, particularly in targeting proteins relevant to BC.

In addition to these applications, WGS and WES play a pivotal role in characterising BC tumours, particularly in delineating spatial heterogeneity and mapping tumour evolution [43]. As an illustration, the utilisation of this technology in BC patients unveiled HER2 gene amplification in circulating tumour cells, despite the absence of HER2 expression in the tumour tissue [43]. This discovery significantly informed treatment decisions. Consequently, WES and WGS hold the potential to elucidate tumour heterogeneity and offer precision medicine approaches for patients.

Furthermore, investigating the spatial heterogeneity of distinct cell populations within BC using WES and WGS has the potential to provide valuable insights into disease progression. By discerning the diverse cellular compositions present within tumours, researchers can uncover novel prognostic markers and genetic factors that contribute to disease recurrence [44]. For instance, the technology facilitated the identification of *ZNF384* overexpression and mutation, which have been linked to a favourable prognosis among BC patients [44]. Moreover, the application of WES revealed that a high tumour mutational burden (TMB) may serve as a prognostic marker, predicting favourable overall survival in well-defined HER2-positive metastatic BC (MBC) patients undergoing conventional HER2-directed treatments and chemotherapy [45]. It is important to note that TMB-high is generally considered a companion diagnostic marker for immune checkpoint inhibitors, as high T < B is associated with increased neoantigen load and potential immune response.

Conventional HER2-directed treatments, such as trastuzumab, exert their effects through mechanisms like antibody-dependent cellular cytotoxicity (ADCC), where antibodies bind to HER2 on tumour cells and recruit immune cells to induce cell death. This immunological mechanism highlights the importance of integrating immune response considerations into treatment strategies. Understanding the spatiotemporal dynamics of the TME is crucial for elucidating the underlying mechanisms driving BC pathogenesis. The recognition of disease-associated genes facilitated by WGS and WES can guide researchers in designing clinical trials and furthering studies, ultimately enhancing our understanding of BC pathogenesis and improving treatment outcomes.

## Challenges of implementing WES and WGS

The integration of WES and WGS into advanced precision medicine holds considerable promise, yet it poses significant challenges in terms of clinical utility. Analysing vast amounts of data, including numerous variables of uncertain significance, contributes to the complexities encountered in clinical applications [46]. Moreover, the absence of standardised procedures and the intricate nature of variant interpretation further exacerbate this challenge [47].

A major obstacle to the widespread adoption of WES and WGS is the associated cost, particularly concerning WES [46]. This issue is of particular concern when implementing these technologies in low- and middle-income countries (LMICs) where resources are limited. For instance, in South Africa, despite the recognition of WES and WGS as powerful tools for deciphering an individual’s genetic makeup, their implementation has been hindered by the high costs [48]. Similarly, in healthcare settings with constrained financial resources, this financial barrier may lead to reluctance in adopting WES and WGS. Consequently, if not addressed adequately, this cost disparity could inadvertently exacerbate health inequities worldwide. Various studies have been carried out to analyse the cost of WES and WGS. The costs of a single test of WES ranged from £382 to £3,592 per patient and for WGS from £1,312 to £17,243 per patient in humans, and from £40 to £487 for bacterial WGS, which confirms the significant financial burden that countries may face when deciding to implement such technologies, unfortunately making it likely for feasible in every patient worldwide [49].

In addition, the ethical implications associated with the use of WES and WGS are substantial. These technologies often lead to the identification of incidental findings (IF), which, despite being unintentional, can cause significant adverse effects, including heightened anxiety among patients and their families [50]. Moreover, managing IF presents a significant challenge for healthcare professionals due to the absence of consensus regarding the definition, analysis, and reporting of such variants to patients and research participants [51].

## Future recommendations

For widespread adoption of WES and WGS, collaborative efforts among researchers, clinicians, policymakers, and bioinformaticians are crucial. These stakeholders must work together to facilitate the integration of WGS and WES into routine clinical practice.

Recent studies have demonstrated the potential of machine learning (ML) algorithms in identifying key genes associated with BC, such as *TXNIP*, *SLC2A1*, *and ATF3*, shedding light on the diseases’ occurrence, development, and prognosis. ML algorithms were also used to construct risk models, stratifying patients into low- and high-risk groups with differing prognoses. Downregulation of *SLC2A1*, identified through this approach, showed promise in suppressing tumour growth, offering a potential therapeutic avenue [52]. This highlights ML’s capacity to integrate omics data and drive personalised medicine, advancing BC diagnosis, prognosis, and treatment.

Furthermore, the integration of single-cell WGS and WES with RNA-seq transcriptomics and slide-DNA/RNA-seq techniques provides a powerful approach to resolve spatial heterogeneity in BC. These technologies aid in constructing comprehensive tumour evolution atlases, elucidating dynamic processes underlying cancer progression. However, cost-effectiveness remains a significant concern, particularly in underdeveloped and developing countries.

To address this issue, the Ultima Genomics UG 100 system, launched on February 6, 2024, represents a significant advancement. This system integrates new sequencing technology with artificial intelligence (AI) to achieve the long-awaited goal of a “$100 genome.” The UG 100 system utilises an open silicon wafer design, featuring hardware upgrades for improved robustness and usability. It also includes enhanced chemistry and analytics for faster run times and superior accuracy. In addition, it incorporates ppmSeq technology, which improves the detection of rare variants, particularly in circulating tumour DNA analysis [53]. PpmSeq (parts per million sequencing) enhances the sensitivity of sequencing, allowing for the detection of low-abundance genetic variants that are crucial for precise cancer diagnostics.

These advancements in sequencing technologies hold promise for accelerating precision medicine and enabling unprecedented population-level studies and disease diagnosis advancements. AI-driven low-cost sequencing technologies, such as the UG 100 system, could significantly expand access to genomic sequencing and improve cancer care globally.

## Conclusion

In conclusion, WES and WGS have significantly influenced BC research and its clinical application. These technologies have offered considerable prospects for personalised treatment, early detection, and ongoing research efforts. Despite encountering challenges in clinical implementation, these advancements have the potential to become indispensable tools in improving BC care and outcomes.
